# Supplementary material for: Characterization of Two Novel Rumen-Derived Exo-Polygalacturonases: Catalysis and Molecular Simulations
Source: Microorganisms. 2023 Mar 16;11(3):760. doi: 10.3390/microorganisms11030760 (PMC10059216; doi:10.3390/microorganisms11030760)
Supplement: Supplementary file 1 [file microorganisms-11-00760-s001.zip › microorganisms-2232197-supplementary.pdf]

Characterization of two novel rumen-derived exo-polygalacturonases: catalysis and molecular simulations

Qian Deng[1,2], Xiaobao Sun[1,2], Deying Gao[1,2], Yuting Wang[3], Yu Liu[4], Nuo Li[1,2], Zhengguang Wang[2], Mingqi Liu[3], Jiakun Wang [1,2], Qian Wang[1,2,*]

[1] Key Laboratory of Molecular Animal Nutrition, Ministry of Education, Zhejiang University, Hangzhou 310058, China

[2] Institute of Dairy Science, College of Animal Sciences, Zhejiang University, Hangzhou 310058, China

[3] Key Laboratory of Marine Food Quality and Hazard Controlling Technology of Zhejiang Province, College of Life Sciences, China Jiliang University, Hangzhou, 310018, China

[4] College of Life Sciences, Zhejiang University, Hangzhou 310058, China

[*] Correspondence author: Tel: +86-0571-88982389

E-mail address: Emirate14@zju.edu.cn

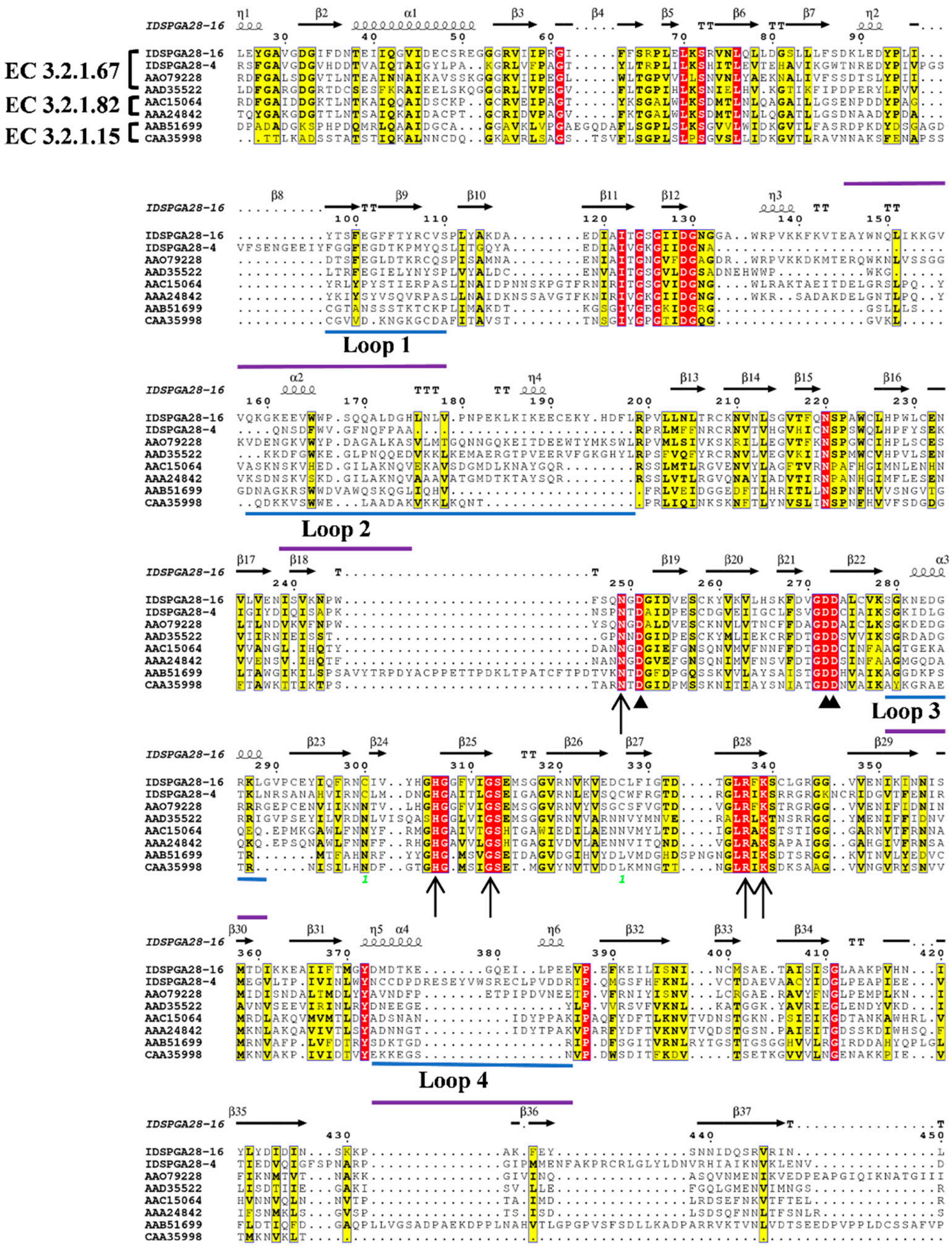

Figure S1 Amino acid alignment of bacterial exo-polygalacturonases. Three conserved catalytic residues (D251, D272, D273) are highlighted by triangles and five residues thought to participate in substrate-binding are indicated by arrows. Four key loops involved in substrate binding and catalysis are underlined in blue (IDSPGA28-16) or purple (IDSPGA28-4).

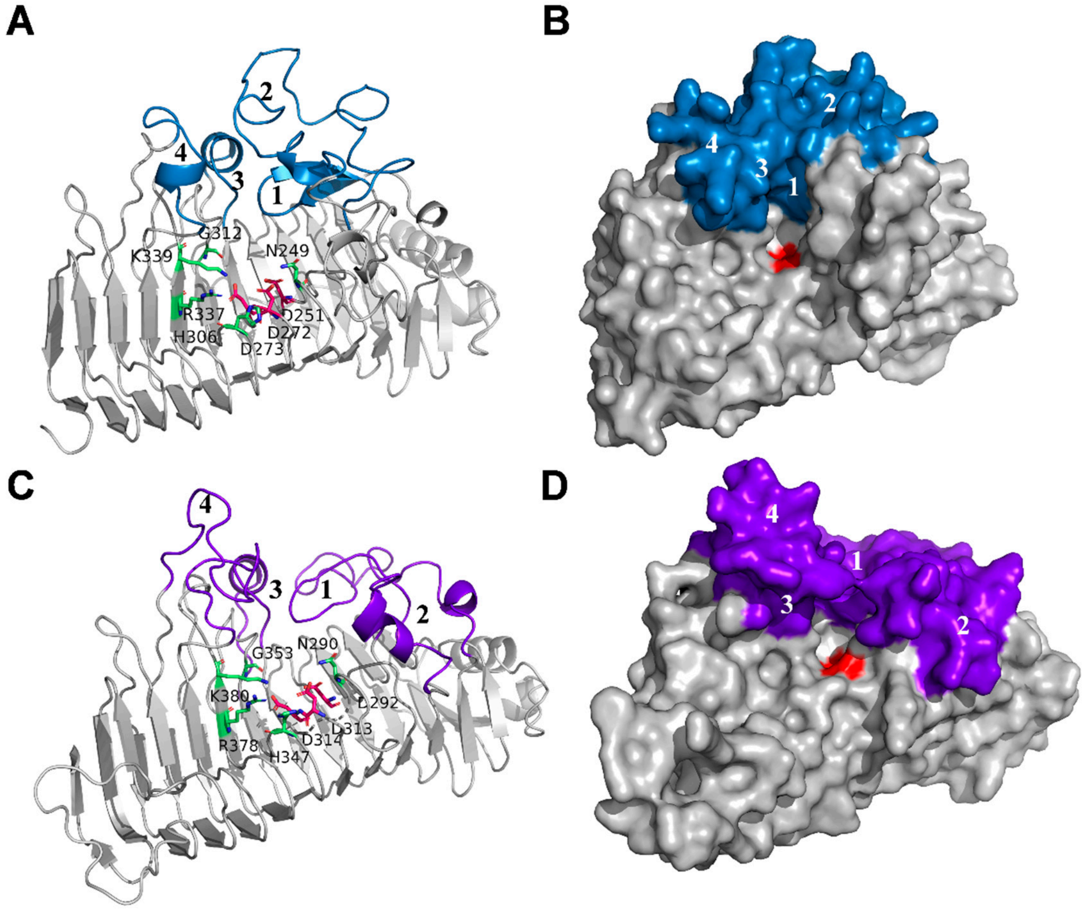

Figure S2 Predicted three-dimensional structure and hydrophobic surface of IDSPGA28-4 (A-B) and IDSPGA28-16 (C-D). Exo-polygalacturonase from *Thermotoga maritima*; (PDB: 3JUR) served as the template for homology modeling. Eight conserved key residues are depicted as sticks. Four key loops involved in substrate binding and catalysis are highlighted in azure (IDSPGA28-16) or violet (IDSPGA28-4). The proposed catalytic center is indicated in red.

Table S1. Oligonucleotides used in this study.

Primers	Sequence (5' to 3')[1]	Purposes
IDSPGA28-4-*Bam*HI	CCATGGCTGATATCGGATCCATGCGAAAAATAAGGTGTCTTAATC	Amplification of *IDSPga28-4*
IDSPGA28-4-*Xho*I	TGGTGGTGGTGGTGCTCGAGCTTTAGCCCTTCCTGATCGA	
IDSPGA28-16-*Bam*HI	CCATGGCTGATATCGGATCCATGTTTAGAACAATGTATATTAAAGAT	Amplification of *IDSPga28-16*
IDSPGA28-16-*Xho*I	TGGTGGTGGTGGTGCTCGAGAGAATTTCCATATGTATCCTCC	
T7-F	GGGAATTGTGAGCGGATAAC	Validation of transformants
T7-R	CCCCTCAAGACCCGTTTAGA	

[1], restriction sites were underlined and homologous arms were shaded.
